# Automatic Information Exchange in the Early Rescue Chain Using the International Standard Accident Number (ISAN)

**DOI:** 10.3390/healthcare9080996

**Published:** 2021-08-04

**Authors:** Mostafa Haghi, Ramon Barakat, Nicolai Spicher, Christian Heinrich, Justin Jageniak, Gamze Söylev Öktem, Maike Krips, Ju Wang, Siegfried Hackel, Thomas M. Deserno

**Affiliations:** 1 Peter L. Reichertz Institute for Medical Informatics of TU Braunschweig and Hannover Medical School, 38106 Braunschweig, Germany; mostafa.haghi@plri.de (M.H.); ramon.barakat@plri.de (R.B.); nicolai.spicher@plri.de (N.S.); christian.heinrich@plri.de (C.H.); maike.krips@plri.de (M.K.); thomas.deserno@plri.de (T.M.D.); 2 Physikalisch-Technische Bundesanstalt PTB, National Metrology Institute of Germany, 38116 Braunschweig, Germany; justin.jagieniak@ptb.de (J.J.); gamze.soeylev_oektem@ptb.de (G.S.Ö.); siegfried.hackel@ptb.de (S.H.)

**Keywords:** accident and emergency informatics, emergency platform, IoT, IoMT, system design, framework, ISAN

## Abstract

Thus far, emergency calls are answered by human operators who interview the calling person in order to obtain all relevant information. In the near future—based on the Internet of (Medical) Things (IoT, IoMT)—accidents, emergencies, or adverse health events will be reported automatically by smart homes, smart vehicles, or smart wearables, without any human in the loop. Several parties are involved in this communication: the alerting system, the rescue service (responding system), and the emergency department in the hospital (curing system). In many countries, these parties use isolated information and communication technology (ICT) systems. Previously, the International Standard Accident Number (ISAN) has been proposed to securely link the data in these systems. In this work, we propose an ISAN-based communication platform that allows semantically interoperable information exchange. Our aims are threefold: (i) to enable data exchange between the isolated systems, (ii) to avoid data misinterpretation, and (iii) to integrate additional data sources. The suggested platform is composed of an alerting, responding, and curing system manager, a workflow manager, and a communication manager. First, the ICT systems of all parties in the early rescue chain register with their according system manager, which tracks the keep-alive. In case of emergency, the alerting system sends an ISAN to the platform. The responsible rescue services and hospitals are determined and interconnected for platform-based communication. Next to the conceptual design of the platform, we evaluate a proof-of-concept implementation according to (1) the registration, (2) channel establishment, (3) data encryption, (4) event alert, and (5) information exchange. Our concept meets the requirements for scalability, error handling, and information security. In the future, it will be used to implement a virtual accident registry.

## 1. Introduction

In 1957, the World Health Organization (WHO) defined an accident as “an event, independent of the will of man, caused by a quickly acting extraneous force, and manifesting itself by an injury to body or mind” [1]. Introducing “accident and emergency informatics” is one of the latest developments in this field [2], indicating the urgent need for forecasting, preventing, or lowering the impact of accidents and emergencies.

Accidents and emergencies can occur anywhere, anytime, and at any scale in any part of daily living. On a global scale, the WHO provides a surveillance system that detects and evaluates public health events for providing emergency funds, field teams, and materials [3]. On a local scale, accidents and emergencies are detected mainly by witnesses and reported by emergency calls [4].

Road traffic accidents and falls account for the majority of injuries. Each year, 1.35 million and 646,000 people lose their lives due to road traffic accidents [5] and unintentional falls [6], respectively. Accidents cause a burden on victims and public health systems. Moreover, the situation is especially severe in low- or middle-income countries. For instance, 93% of all road traffic deaths and 80% of all deadly falls occur in these countries. The risk of suffering from an accident depends on age: 73% of all road traffic deaths occur among young males, and death rates because of falls are highest among adults older than 60 years [5,6].

Despite technical innovation in the last decades, emergency services still rely on manual alarming and humans operating an emergency hotline. The responder asks the caller for relevant information (“Where?”, “What?”, “Who?”, etc.) and dispatches the according rescue team, e.g., an ambulance. Recently, technological innovations have been developed aiming at automatic alerting. For instance, the European Union established eCall [7]: when a car deploys its airbags, it establishes a phone connection to an emergency hotline and sends a minimum set of data (MSD) before the human operator takes over. The MSD contains the car type, car ID, car energy system, and GPS coordinates. In research, the car as a diagnostic space, i.e., as a way of unobtrusively measuring bio-signals, is the focus of attention [8,9].

Automatic sensors (e.g., fire and smoke detectors) have a long history of detecting in-home emergencies. However, they are limited to the monitoring of the environment but exclude individuals. Individuals’ accidents (e.g., falls) are reported manually [10]. For example, bracelets or necklaces are equipped with a button, which will establish a phone connection with an emergency hotline once pressed [11]. The recent trend towards smart wearables further strengthens developments in this direction [12,13,14].

However, the current state of emergency alerting has several bottlenecks and shortcomings. The parties involved in the rescue operation, such as the rescue services and emergency departments in medical centers, often use isolated information and communication technology (ICT) systems. The lack of interoperability between the ICT systems implies that information is passed verbally from one party to another, or on paper. During this process, important information is at risk of being lost, transmitted incorrectly, or delayed.

Recently, we proposed the International Standard Accident Number (ISAN) [15] to facilitate machine-to-machine (M2M) communication with the Internet of (Medical) Things (IoT, IoMT). The ISAN is a unique identifier for accidents and emergencies and makes fully automatic alerting feasible. It contains all relevant information about an event (i.e., time, location, altitude). It serves as a common identifier to link data from the isolated ICT systems involved in the early rescue chain: alerting, responding, and curing facilities.

In this work, we propose a conceptual design of an emergency communication platform for ISAN-based data linkage. In this design, we introduce a system architecture including all necessary blocks and components for communicating between different ICT systems. The linked data may include site or floor maps, vital signs, dash-cam videos, rescue data sheets, and many others. We provide an experimental proof-of-concept and validate the functionality of the platform using the Session Initiation Protocol (SIP) [16]. Our aims are threefold: (i) to enable semantic interoperable and secure data exchange between the isolated systems, (ii) to avoid data misinterpretation, and (iii) to integrate additional data sources.

## 2. Related Work

### 2.1. Accident Data Acquisition in Smart Environments

Nowadays, IoT devices cover smart living (e.g., smart homes) and smart mobility (e.g., smart vehicles, smart wearables). Using IoT sensors and platforms can acquire and process accident data [17]. Integrating sensors in the facilities where accidents can happen, we obtain more objective and precise data during or even before the adverse event.

#### 2.1.1. Smart Homes

Due to unfamiliarity with the accident location, rescue personnel may need more time to identify the fire location and the victims to be rescued. The IRiS project researched how a smart home integrated with different sensors and actors can support rescue operations [18]. Once the smart home detects fire, the static information (address, registered resident, building layout/plan) and dynamic information (triggered smoke detector, live video, and movement sensor data) can be transferred to an authorized agent. The agent obtains sufficient information to evaluate the alarm and dispatches appropriate firefighters. Schultz et al. developed a tablet application for firefighters to access the smart home data on the way to the fire site. The application can navigate the accident location, provide access to the accident building, demonstrate the danger points in the building, and, when necessary, it can switch on a light or roll shutters to indicate the position of missing persons or the origin of the fire [19].

#### 2.1.2. Smart Vehicles

Fogue et al. designed and implemented an on-board system for automatic accident detection [20]. The system captured sensor data of the most relevant variables that can characterize the severity of the accidents, including vehicle speed, the type of vehicles involved, the impact speed, and the airbag status. The data collected are packed and forwarded to a remote-control unit through a combination of vehicle-to-vehicle (V2V) and vehicle-to-infrastructure (V2I) wireless communication. Furthermore, they developed a Knowledge Discovery in Databases (KDD) process to estimate accident severity.

#### 2.1.3. Smart Wearables

Recently, smart wearables (e.g., watches) have attracted great interest for automatic fall detection [21]. As an example, the Apple Watch (Series 4, Apple, Los Altos, CA, USA) generates an alert on the display when it detects a fall. However, this remains tuned whether the user responds to the message. If no action is taken and the watch does not detect any movement, an emergency call containing the GPS coordinates is made [22]. To reduce the number of false alerts, Maglogiannis et al. propose a hybrid approach pairing a Pebble smartwatch (Perbble Technology, Redwood, CA, USA) and an Android smartphone [23]. In the same manner, Vilarinho et al. connected a LG watch (G R1, LG Electronics, Seoul, Korea) and an Android Samsung Galaxy (S3, Samsung Electronics, Seoul, Korea). Rakesha reports falls only if confirmed by threshold-based analysis of the signals from the built-in accelerometers of both devices [24].

### 2.2. Emergency Communication Platforms

Nowadays, numerous emergency call control centers in Europe still use legacy telecommunication networks to receive and process emergency calls. Emergency calls from IP networks are translated within the network [25]. However, legacy telecommunication networks are outdated and will be replaced by IP-based technologies. This will also apply to emergency communication services. The European Emergency Number Association (EENA) has specified a long-term definition of a European emergency services architecture [26], which is primarily focused on bringing together current heterogeneous telecommunications systems, such as legacy telecommunication networks, IP-based voice services, and the dedicated emergency services internet protocol network (ESInet) [27].

To achieve a suitable emergency service architecture based on IP, the National Emergency Number Association (NENA) and the European Commission provide reference architectures for implementing the so-called Next Generation architectures for emergency services (NG112/NG911) based on Voice over IP (VoIP) technologies [27,28]. Various research projects and initiatives, such as EMYNOS [17], NEXES [29], or the EENA NG112 Project [30], have started to develop such NG112 Emergency Platforms based on the aforementioned reference architectures.

In general, NG112 architectures are not only expected to standardize the communication infrastructure (ALL-IP). The rudimentary data exchange, which can be easily implemented in IP networks, offers several potential advantages over voice calls, e.g., more efficient and less error-prone data gathering and faster provision of situation-specific information [31,32,33]. Various research projects are trying to expand the amount of data within emergency communication by integrating various information such as data provided by IoT devices and smart systems [17,29,34].

The location of help seeker(s) is the most significant information for emergency call centers. Advanced mobile location (AML) is a protocol supported with ETSI TS 103 625 as a technical specification developed at the European level. AML has been developed for transmitting the accurate location of a caller to the emergency center, advocated by EENA [35]. In an emergency call, the AML-enabled smartphone transmits the accurate location data of the caller using SMS and/or HTTPS from the smartphone to the emergency call center. Since December 2020, the European electronic communication code forces all member states to use handset-derived locations to track emergency callers’ locations [36]. Starting from March 2022, all smartphones sold in the European market have to offer the possibility of handset-derived location information of the caller to the emergency services [36].

### 2.3. Data Linkage across Systems

Data linkage is a necessary procedure in data integration. Without unique identifiers (e.g., ISAN), deterministic and probabilistic methods apply. Deterministic linkage uses a predefined algorithm to combine variables and generate a unique identifier, namely a linkage key [37]. A pair of data records are linked once their linkage keys match. Probabilistic linkage applies to cases where unique identifiers are not available or linking variables are not as accurate, stable, or complete as required for deterministic linkage [38]. Then, the link is determined to achieve a close approximation to unique identification through several linking variables. Each of these variables only provides a partial link. In combination, the link is sufficiently accurate. Both methods have been applied to link pre-hospital accident data and the datasets from emergency departments [39].

#### 2.3.1. Deterministic Linkage

Clark et al. conducted a study to link records of the patients conveyed by a single emergency ambulance service to thirteen emergency departments in the UK from 2012 to 2016 [40]. First, they collect de-identified patient record data from the emergency department. To generate the linkage key, they chose two event identifiers that were consistent across systems (the emergency ambulance service and the emergency department) as the linking variables, i.e., ambulance incident number and vehicle shift number (call sign). There are two strategies: combining (i) the ambulance incident number with the accident date; (ii) the vehicle shift number with the date and time.

#### 2.3.2. Probabilistic Linkage

Govindarajan et al. linked county-wide patient-level ambulance data with emergency department and patient discharge data [41]. They implemented a probabilistic matching algorithm. The variables, including the patient’s transport/admission date, date of birth, race, sex, county of residence, and destination hospital, set the basis of the linkage model. The model calculates the probability that a pair of records is a true match based on the linkage variables’ agreement/disagreement patterns (match probability ≥ 0.8).

## 3. Requirement Analysis

Rescue operations are complex and time sensitive. Introducing M2M communication in this field, we aim at reducing processing time and system faults and avoiding human factors in the loop. We interconnect all ICT systems in the alerting, responding, and curing facilities. To secure semantic interoperability, we derive particular requirements.

### 3.1. System Architecture

Each involved ICT system has distinct hardware, software, location, and services. This leads to a multitude of information that a single system cannot manage. Therefore, all systems need to register at the central emergency communication platform, storing the properties of each connected ICT system. Hence, the developed architecture is a star network with the emergency communication platform being the central hub and all other ICT systems acting as a host.

### 3.2. Data Exchange

Either the data provider or the data consumer initiates the data exchange between the central platform and the connected ICT systems. While the platform needs to “pull” data from the connected systems, e.g., for administrative purposes, the systems also need to “push” data to the platform, in particular in case of an emergency. Regarding the data format, we need to ensure technologic, syntactic, and semantic interoperability. While internet protocols maintain technical interoperability, we lack international classifications, terminologies, and standards for semantically interoperable information exchange during rescue operations [42].

### 3.3. Safety and Security

Data exchange in the early rescue chain inevitably transmits personal and sensitive health data. Therefore, it is necessary to secure the data and to ensure integrity and confidentiality [43]. Thus, data exchange should be aligned with the national (e.g., security) and international regulations, such as the German Federal Office for Information [44] and SOGIS-2018, SOGIS-2020 [45], which recommend encryption and digital signature in electronic health cards and digital health infrastructures. Additionally, data encryption and transmission should have minimal latency. Due to the sensitivity of the operation in an emergency, the availability of the system must be backed up against hardware failures and loss of connectivity.

## 4. System Specification

### 4.1. Architecture

**Figure 1:**
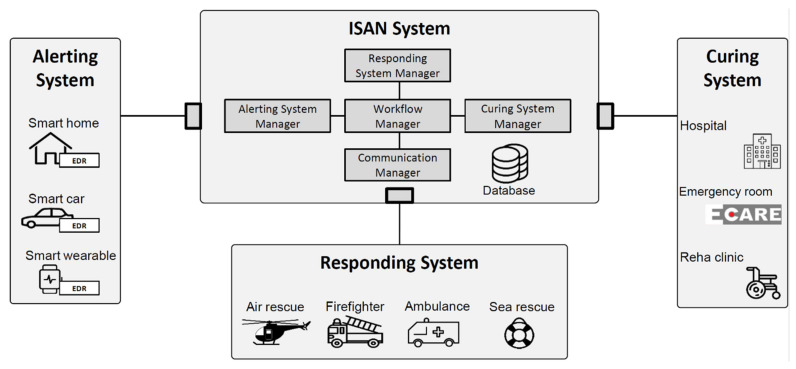
System architecture of the ISAN emergency communication platform. The ISAN system is triggered by an alerting system, handled by ASM, and specifies respective responding and curing systems by RSM and CSM, respectively. It manages the communication for data transfer by WFM and ComM.

To fulfill the requirements, we propose an emergency communication platform to link data across pre- and in-hospital stages of the early rescue chain. We compose our architecture of the alerting, responding, and curing system, and the ISAN core system (Figure 1). The alerting system represents the ICT system at the accident site. It collects relevant data and generates an ISAN-based alert upon an accident. The responding system is represented by the first responder’s ICT system and refers to the rescue team tuned upon an event. The curing system refers to ICT systems in hospitals and emergency rooms. As the coordinator of the isolated ICT systems, the ISAN system monitors the ICT system status, responds to alerts, and establishes secure platform-based communication channels between the systems.

#### 4.1.1. ICT Systems

An alerting system is a sensor-enhanced living environment, which could be a smart home [19], a smart car [9], or a smart wearable [46]. We aim to incorporate potential technologies in the rapidly evolving field of IoT/IoMT. The alerting system continuously monitors the environment. As soon as an adverse event is detected, the system collects relevant data, generates the ISAN, and initiates the communication by sending an alert message to the ISAN system. The event data recorder (EDR) stores relevant data. The alerting system can actively transfer a digital accident report (DAR) or passively provide the DAR, if requested by another ICT system.

The first responding entity in the rescue chain (e.g., rescue service, firefighters) runs the responding ICT system. It receives alerts from the alerting system via the ISAN system. The responding system parses time and location from the ISAN in a human-readable manner. This information facilitates dispatching the right rescue team to the right location at the right time—minimizing delays and avoiding human errors.

The medical institutions receiving injured subjects operate the curing ICT systems, which receive personal data, medication, or vital signs before the subject’s delivery. A curing system can actively request the subject’s pre-hospital data, including DAR and EDR of the alerting system.

#### 4.1.2. ISAN System Components

The ISAN system coordinates the communication between all ICT systems and enables information exchange between the isolated alerting, responding, and curing systems.

Apart from a database and interfaces to the other systems, it consists of several components: a workflow manager, a communication manager, and ICT system managers (Figure 1). We secure all interfaces between these ICT systems with encryption. For secured data exchange, we tense an encryption method based on the elliptical curve and the Curve25519, a standard for digital signatures and key exchange protocols. We use this encryption technique because: (i) its mechanism is faster than other security algorithms such as River–Shamir–Adleman (RSA); particularly, it outperforms RSA for higher level of security, (ii) in alerting systems with restricted computational resources (e.g., wearables), Curve25519 as an efficient and light-weight encryption mechanism is preferred, and (iii) it is recommended in guidelines of the German Federal Office for Information Security (Bundesamt für Sicherheit in der Informationstechnik) for applications in the health sector [47].

We introduce the alerting system manager (ASM), the responding system manager (RSM), and the curing system manager (CSM). Any ICT system registers itself to the corresponding system manager. These three components address integration of additional data sources. The according system manager maintains a database tracking the status of all registered ICT systems. Furthermore, it provides data required by the workflow manager during communication. For instance, the RSM maintains the information about the locations and the network layer addresses of the responding systems, their areas of responsibility, available rescue services (e.g., specialized equipment, personnel). Based on this information, the RSM can identify the most suitable responding system for an accident given the accident location (included in its ISAN), and provide the destination address to route the alert message accordingly. With a similar procedure, the CSM determines the most suitable curing system for an accident, considering the accident data in the DAR and the registered information of the curing system (e.g., the capacity of the emergency room/intensive care unit, availability of the stroke unit).

As the central node, the workflow manager (WFM) orchestrates all messages and system interactions. It processes messages from all ICT systems. The WFM also verifies the messages to protect the ISAN system from fake alerts, invalid or unauthorized data requests, and cyber-attacks. Depending on the message type, the WFM asks for further data from an ICT system manager and negotiates proper communication channels with the communication manager (ComM). The WFM is also responsible for the appropriate system operation, including load balancing and error handling. It prevents call drops by forwarding the call to an alternative responding/curing system.

If two ICT systems transfer data, the ComM handles the communication between the two systems appointed by the WFM from the communication protocol perspective. Then, it initiates the platform-based communication by informing the peers and taking over the role of connection establishment, data exchange routing, and connection termination. The sequence workflow and mechanism of WFM and ComM, incorporating with ICT manager, avoids miscommunication, mislinkage, and misinterpretation of data.

### 4.2. Interaction

#### 4.2.1. System Registration

An ICT system has to register with the ISAN system before creating alerts and exchanging information with other systems. Registration and authorization is one component of security to prevent faked alerts or data requests. However, registration regulations have to ensure that no unauthorized system registers. During the registration process, the system receives an ID (SIP address). This ID is stored in the databases of the corresponding system managers. Any request without valid ID is blocked and remains suspended. The system managers further collect basic information (Table 1). This information is in need to route messages to the correct destinations.

We define the system status as time-based, and it can expire. The system managers periodically send *keep-alive* messages to the registered system and receive its response. This maintains an accurate overview of all registrations and ensures that data is not left in the database for too long. This is especially important for responding and curing systems. If emergency calls would first be forwarded to a responding or curing system, which is disconnected, valuable time is wasted.

**Figure 2:**
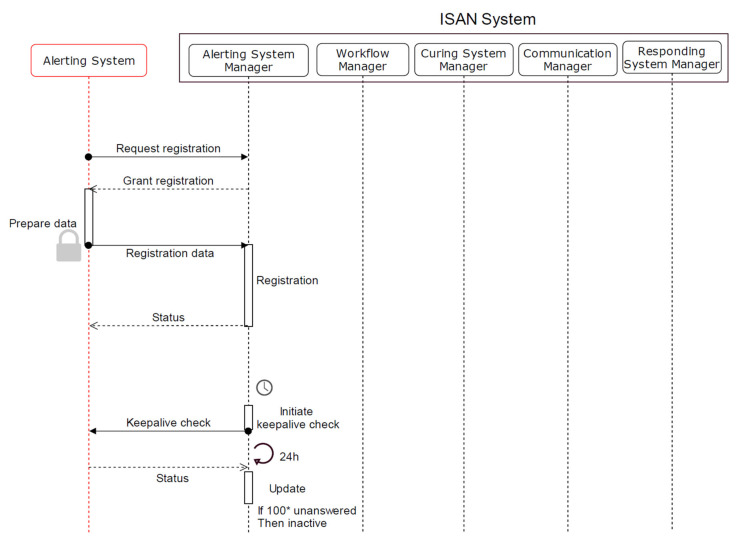
Registration of alerting system.

The registration process involves the alerting system and the ASM only (Figure 2). All data exchange is encrypted.

1. To initiate the process, the alerting system sends a registration request.
2. On a valid request, the ASM approves the registration by dedicating an ID and storing it.
3. The alerting system prepares the required data, including the standards in use for ISAN generation, a list of alert types, and a unique identifier of the alerting system.
4. The ASM receives the data, writes it into the database, and sends a confirmation message to the alerting system.
5. The ASM regularly checks the state of the alerting system by sending a *keep-alive* message. If the alerting system does not respond several times, the ASM updates the alerting system status as *inactive*.

The registration of responding and curing systems follows the same procedure. It differs in the registration data and the keep-alive check (Table 1).

#### 4.2.2. Alerting and M2M Communication

We propose a system architecture that supports platform-based communication. Collecting accident and emergency data starts with the system registration even before an adverse event occurs. The alerting system generates the ISAN as a unique token of a certain accident. Using this ISAN, the data provider or consumer can initiate M2M communication. Furthermore, data can be transferred simultaneously between, for instance, an alerting and a responding system as well as the alerting and curing system.

We have successfully tested the 2.4 GHz frequency band. For file transmissions, we use the session description protocol (SDP) that is incorporated with SIP. In case of data loss, the data can be re-requested.

**Figure 3:**
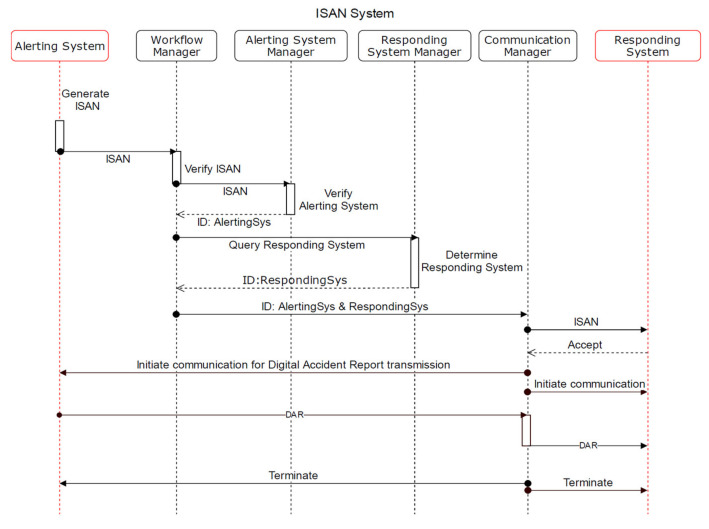
Alerting system actively transfers data to responding system.

**Figure 4:**
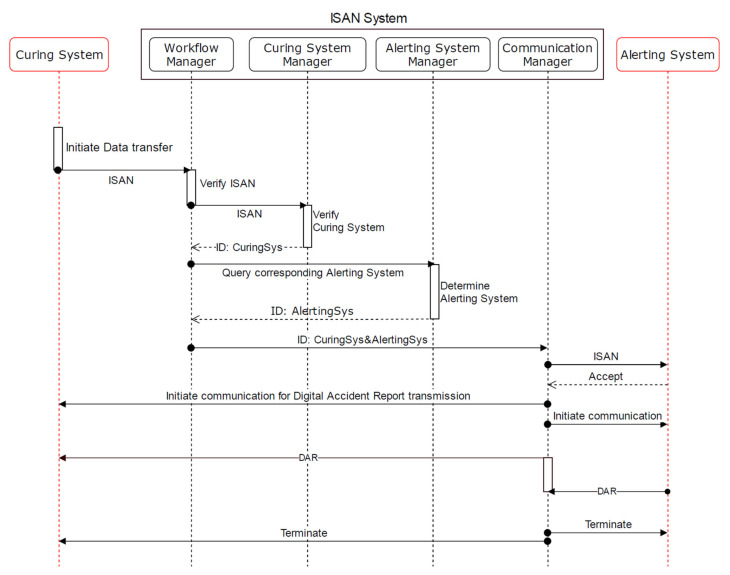
Curing system request data from alerting system.

We demonstrate two use cases: (i) the alerting system transfers data to responding system (Figure 3); (ii) the curing system requests data from the alerting system (Figure 4).

The workflow of both scenarios is similar. The difference lies in the starting and ending point. The following description corresponds to Figure 3.

1. In case of an accident, the alerting system generates the ISAN and sends it to the WFM.
2. The WFM verifies the ISAN and forwards it to the ASM to further verify the alerting system, where the ASM will check the status stored in the supporting database. The ASM returns the ID of the alerting system to WFM.
3. The WFM sends a query to the RSM to determine a suitable responding system. The RSM searches its supporting database and returns the ID to an appropriate responding system.
4. The WFM forwards both IDs (alerting and responding system) to the ComM.
5. The ComM informs the responding system, including the ISAN, that an accident occurred, and the alerting system needs to transfer accident data, i.e., the DAR.
6. The responding system accepts the request.
7. The ComM establishes the communication with the alerting and responding system.
8. The alerting system transfers the DAR to the responding system via the ComM.
9. The ComM terminates the communication with both systems.

### 4.3. Proof of Concept

To provide a proof of concept, we have implemented the system architecture as a virtual testbed, which we host on an off-the-shelf server. Based on the Apache License 2.0, we run all components in a separate virtual container (Docker Container, available online: https://www.docker.com/ (accessed on 31 May 2021)) and deploy in an orchestration service providing automatic management (Kubernetes, available online: https://kubernetes.io/ (accessed on 31 May 2021)).

We use SIP to distribute the ISAN and establish communication between the ICT systems. The SIP is an application layer protocol mainly utilized for—but not limited to—voice over internet protocol (VoIP) architectures. It is a signaling protocol primarily for establishing, controlling, and terminating communication sessions [48]. We customized the open-source SIP software (Kamailio, available online: https://www.kamailio.org/ (accessed on 31 May 2021); GPL license).

#### 4.3.1. ISAN System Component Implementation

We realize all managers as SIP registrar servers, which maintain a database to store information of the ICT systems. A registrar is an SIP endpoint that manages the registration of clients, i.e., ICT systems. It records the registration data (Table 1) from the client and provides an essential tool to locate communication peers on the network. For each ICT system, an identifier (SIP URI) and the IP address are stored in the database.

We realize the WFM as a SIP routing proxy that processes the SIP messages. In case of an adverse event, the responding system extracts the alert message, including the ISAN from the SIP header, and determines the SIP address of the responding or curing system via the system manager.

We also realize the ComM as a SIP proxy that monitors the negotiation of the SIP session between the peers. The ComM forwards the message to the desired receiver, according to the SIP address provided by the workflow manager, and informs the responding system about the alert. The SIP message body contains information about which media types are supported by the alerting system and which protocols are used for this purpose. If a requested ICT system does not respond or cannot process the requested media type, the ComM reports to the WFM that is unable to establish the communication. Then, the WFM identifies and replaces the next responding or curing system, which is the closest located system to take over. The WFM updates the process and returns it to the ComM to retry establishing the communication.

#### 4.3.2. Communication

During the registration, each system is assigned a unique ID (SIP address) which is stored in the databases of the corresponding system manager. For registering the system or client device, a registration message (SIP REGISTER with authentication) must be sent to the corresponding ICT system manager. This message includes the identifier, which is bound to the IP address. This SIP REGISTER message is sent periodically as keep-alive messages.

We realize inter-system communication by SIP INVITE messages that contain an ISAN. In the alerting message, the SIP address sip: alert@isan.de is specified as the recipient because the actual receiver needs to be determined by the ISAN system. Given the ISAN, the WFM requests the RSM to obtain the SIP address of the responsible (or closest available) system. We forward the SIP addresses of the alerting and responding systems and the alert message to the ComM. Then, ComM requests establishing the communication with both systems. If the responding system accepts the communication, it replies with an accept message (SIP OK). The ComM establishes the parallel communication with the alerting and responding system and delivers the data (e.g., DAR) to the responding system. We use platform-based communication for the actual data transfer. After successful transfer, ComM terminates the communication with both systems and closes the channel (SIP OVER).

To support secure data transmission, we apply Curve25519, which is an efficient security standard today. Encryption and decryption rely on the Diffie–Hellman method with a shared secret key, generated from a combination of the public key of one side and the private key of the other side [49]. For this purpose, the public keys must be exchanged within the initial alert message (SIP INVITE). This process is also called “X25519 key exchange” [50]. The signature process based on this curve is called Ed25519. When the asymmetric encryption is used, the exchanged public keys in X25519 will be validated by Ed25519 for contributory behavior [51].

We have tested our approach by simulating an event. We have located an ultrasonic distance sensor (HC-SR04, Sparkfun Electronics, Boulder, CO, USA) on the frame of a door in smart home to detect a person crossing the line in/out of the room. We consider such an activity as an event due to the required instant response time by the sensor and system. We connected the sensor to a board based on a processing unit (AVR 16F887, Microchip, Chandler, AZ, USA) for processing and event detection. Upon detecting such an event, the board sends a trigger to the central single-board computer (Raspberry Pi 4B, Raspberry Pi Foundation, Cambridge, UK), which pushes an internal interrupt with the highest priority to generate the ISAN. The exemplary generated ISAN reads as follows; for syntax and semantics, see [15]:

The Raspberry Pi 4B delivers the time (20210528T154942 + 0200). We use a GPS coordinate module (Breakout board MTK 3339, Adafruit, New York, NY, USA) (+52.2734761 + 10.5242842) to simulate the more general mobile setting (e.g., car accident). The MAC address of our Raspberry Pi delivers the unique identifier of the ISAN (e4:b9:7a:6a:fb:50).

**Figure 5:**
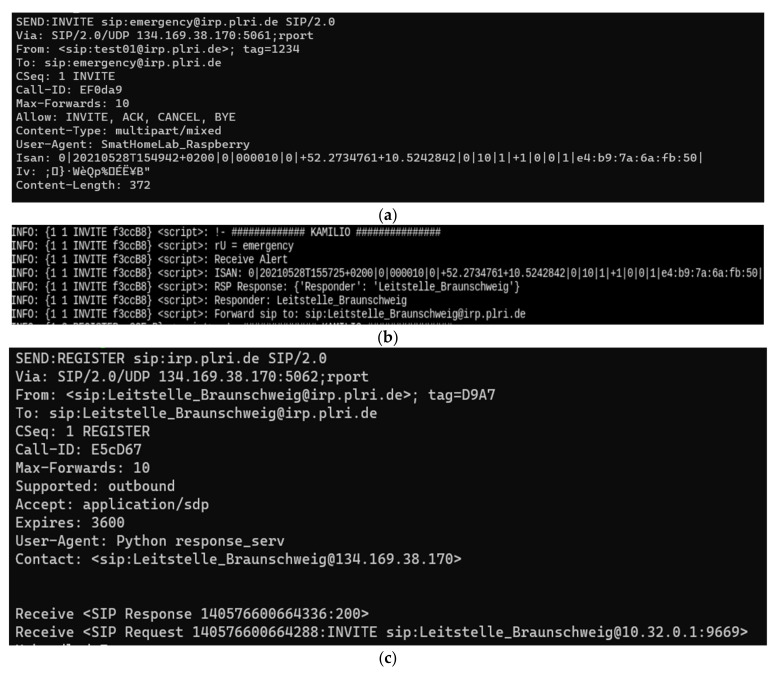
Logs from ISAN system communication with the alerting system (**a**), internal communication (**b**), and the responding system (**c**) to receive, process, and transfer the ISAN upon an emergency, respectively.

We set up a virtual machine to simulate the responding system. We implemented the responding systems according to the rescue team stations in the city of Braunschweig, Germany. We registered all to the ISAN system (alerting system ID: EF0da9 and responding system ID: E5cD67). In the event, the alerting system, i.e., SmartHomeLab_Raspberry generates an ISAN and sends it to the ISAN system (Figure 5a). The ISAN system processes the token and extracts the event’s location and the responsible control station (German: Leitstelle) (Figure 5b). The responding system accepts the invitation and acknowledges the availability to start the rescue operation (Figure 5c).

## 5. Discussion

Using the ISAN, our platform allows data linkage of pre- and in-hospital health data within the entire rescue chain. We provide interfaces to the ISAN system for the alerting, responding, and curing systems such that different technologies and applications can communicate with each other. While several ICT system managers interconnect individual ICT systems and monitor their availability, the ComM moderates their communication based on the stored data (e.g., responsibility of the responding system (local area), availability of the curing system (special treatments)).

We treat all events equally, but we consider the type of event to select the appropriate responding and curing systems. Extending the International Classification of Diseases (ICD 11), a standardized terminology for the type of event is needed [42].

There is related work on accident data acquisition in smart environments [18,20]. In contrast, the ISAN communication platform intends to establish secured data transfer between authorized agents. According to the ISAN paradigm, the data linkage key is generated in place, i.e., at the time of the event. This increases the accuracy of accident data matching as compared to the typical approaches, which rely on keys that are generated afterwards or centralized [40,41].

We meet requirements for scalability and error handling (e.g., if an ICT system or component does not respond) by dividing the emergency communication into individual components, which we run in parallel. As we consider data security in healthcare as very important, our encrypted connection ensures that sensitive data can neither be read by external parties nor by the emergency platform itself. Additionally, our registration procedure ensures that only trustworthy systems are included. In the case of any misuse, such as fake emergency calls or attacks by hacked systems, the questionable system can be identified and, if necessary, blocked.

As we use standardized communication protocols, such as SIP, we can interact with other information systems in healthcare and rescue. Since the NG112 architecture also uses SIP, future NG112 emergency call platforms can be integrated into our system architecture by providing special interfaces for NG112 emergency calls [52]. However, the platform is also adaptable to other protocols on demand.

The ISAN system may also serve as a complement to the NG112. The NG112 platforms and reference architectures are designed for human-to-human communication by using VoIP technologies. In contrast, the ISAN system is designed for M2M communication only. Furthermore, the NG112 architecture considers emergency call centers only. In contrast, the proposed ISAN system considers all ICT systems involved in a rescue operation [53].

In addition, we consider the ISAN system as a complementary international standard to the emergency and public health system. The ISAN system can contribute to public health by integrating into emergency centers and commercial emergency service providers (e.g., ISE Cobra 4, Rescue track). This boosts the intercommunication for exchanging information and services.

When handling automatically generated alerts without any human in the loop, we need to cope with false alerts, too. However, the focus of this work is on linking the ICT systems rather than on verifying generated alerts. Currently, we assume the alert is valid to initiate the responding procedure. There is ongoing research on sensor fusion from all the WHOQOL domains to make event detection more accurate [46].

In the future, we will integrate a virtual accident registry into the ISAN system. The registry will collect accident and emergency data for secondary usage in either research or quality assessment of emergency care. Data sources can be all ICT systems participating in the rescue operation, and the ISAN will be used as the key identifier of an accident.

Thus far, we have implemented security mechanisms. In the future, we will deploy an intrusion detection system (IDS) to identify active attacks. The IDS will continuously scan the network to prevent malicious traffic from entering the system.

Furthermore, we will address the scalability of the ISAN system by adding several smart homes, cars, and wearables and simultaneously create events. Furthermore, we plan to integrate the ISAN system into the emergency service providers in the city of Brunswick, Germany.

This is a local scale. Increasing the scale will also allow us to overcome the eventual unavailability of the required responding system by requesting national or even international assistance.

## 6. Conclusions

We have proposed an ISAN-based emergency communication platform, which connects the ICT systems involved in the early rescue chain. As a unique token, the ISAN is broadcasted in the system, yielding data linkage of separated silos in alerting, responding, and curing instances. Our system supports platform-based communication of further information to improve rescue operations and medical treatment. Our approach enables comprehensive data transfer with minimal delay. New devices and systems can be integrated easily, supporting further applications.

## Figures and Tables

**Table 1:** Registration dataset for ICT systems.

Defaults	Alerting System	Responding System	Curing System
Dataset	Used standards for ISAN generation List of alert types Unique identifier	Supported ISAN standards Location Area of responsibility Provided rescue services Unique identifier	Supported ISAN standards Location Area of responsibility Provided medical services Unique identifier
Keep-alive frequency	24 h	10 min	10 min
Allowed times being unresponsive	100	3	3

## Data Availability

Data sharing not applicable.

## References

[1] World Health Organization (1957). Accidents in Childhood: Facts as a Basis for Prevention, Report of an Advisory Group.

[2] Accident & Emergency Informatics—IMIA A&EI WG—IMIA https://imia-medinfo.org/wp/accident-emergency-informatics-working-group/.

[3] EWARS—Improving Early Detection and Prompt Response to Acute Public Health Events|WHO|Regional Office for Africa https://www.afro.who.int/news/ewars-improving-early-detection-and-prompt-response-acute-public-health-events.

[4] Møller T.P., Ersbøll A.K., Tolstrup J.S., Østergaard D., Viereck S., Overton J., Folke F., Lippert F. (2015). Why and when citizens call for emergency help: An observational study of 211,193 medical emergency calls. Scand. J. Trauma Resusc. Emerg. Med..

[5] Ivers R., Brown K., Norton R., Stevenson M. (2016). Road traffic injuries. International Encyclopedia of Public Health.

[6] Falls [Internet]. https://www.who.int/news-room/fact-sheets/detail/falls.

[7] Li Y., Nader M., Liu J.Q. In-Vehicle system design for the European Union emergency call. Proceedings of the IEEE International Conference on Electro Information Technology.

[8] Leonhardt S., Leicht L., Teichmann D. (2018). Unobtrusive vital sign monitoring in automotive environments: A review. Sensors.

[9] Wang J., Warnecke J.M., Haghi M., Deserno T.M. (2020). Unobtrusive health monitoring in private spaces: The smart vehicle. Sensors.

[10] Thilo F.J.S., Hahn S., Halfens R.J.G., Schols J.M.G.A. (2019). Usability of a wearable fall detection prototype from the perspective of older people—A real field testing approach. J. Clin. Nurs..

[11] Fajingbesi F.E., Olanrewaju R.F., Pampori B.R., Khan S., Yacoob M. (2017). Real time telemedical health care systems with wearable sensors. Asian J. Pharm. Res. Health Care.

[12] Silva de Lima A.L., Smits T., Darweesh S.K.L., Valenti G., Milosevic M., Pijl M., Baldus H., de Vries N.M., Meinders M.J., Bloem B.R. (2020). Home-based monitoring of falls using wearable sensors in Parkinson’s disease. Mov. Disord..

[13] Haghi M., Thurow K., Stoll R. (2017). Wearable devices in medical internet of things: Scientific research and commercially available devices. Healthc. Inform. Res..

[14] Amiri A., Peltier N., Goldberg C., Sun Y., Nathan A., Hiremath S.V., Mankodiya K. (2017). WearSense: Detecting Autism stereotypic behaviors through smartwatches. Healthcare.

[15] Spicher N., Barakat R., Wang J., Haghi M., Jagieniak J., Öktem G.S., Hackel S., Deserno T.M. (2021). Proposing an International Standard Accident Number for interconnecting information and communication technology systems of the rescue chain. Methods Inf. Med..

[16] SIP Protocol—VoIP-Info https://www.voip-info.org/sip/.

[17] Markakis E.K., Lykourgiotis A., Politis I., Dagiuklas A., Rebahi Y., Pallis E. (2017). EMYNOS: Next generation emergency communication. IEEE Commun. Mag..

[18] Schultz A., Lamers C., Koch R., Lüke R., Sauerland T. (2020). IRiS—Intelligente Rettung im SmartHome. VFDB Z. Forsch. Tech. Manag. Brand..

[19] Wang J., Spicher N., Warnecke J.M., Haghi M., Schwartze J., Deserno T.M. (2021). Unobtrusive health monitoring in private spaces: The smart home. Sensors.

[20] Fogue M., Garrido P., Martinez F.J., Cano J.C., Calafate C.T., Manzoni P. (2014). A system for automatic notification and severity estimation of automotive accidents. IEEE Trans. Mob. Comput..

[21] González-Cañete F.J., Casilari E. (2021). A feasibility study of the use of smartwatches in wearable fall detection systems. Sensors.

[22] Use Fall Detection with Apple Watch—Apple Support https://support.apple.com/en-gb/HT208944.

[23] Maglogiannis I., Ioannou C., Spyroglou G., Tsanakas P. (2014). Fall detection using commodity smart watch and smart phone. IFIP Adv. Inf. Commun. Technol..

[24] Rakhecha S., Hsu K. Reliable and secure body fall detection algorithm in a wireless mesh network. Proceedings of the BODYNETS 2013—8th International Conference on Body Area Networks, ICST.

[25] Patel M., Kumar M., Gupta P., Bhatnagar J. Determination of PSAP and routing of emergency calls in IP multimedia subsystem. Proceedings of the ANTS 2017—11th IEEE International Conference on Advanced Networks and Telecommunications Systems.

[26] NG112 E Next Generation 112 Long Term Definition Standard for Emergency Services Document (Version 1.1) [Internet]. https://eena.org/wp-content/uploads/Next-Generation-112-Long-Term-Definition-Standard-For-Emergency-Services.pdf.

[27] Sedlar U., Winterbottom J., Tavcar B., Sterle J., Cijan J., Volk M. (2019). Next generation emergency services based on the Pan-European Mobile Emergency Application (PEMEA) protocol: Leveraging mobile positioning and context information. Wirel. Commun. Mob. Comput..

[28] NENA NENA Functional and Interface Standards for Next Generation 9-1-1 Version 1.0 (i3) [Internet]. https://www.nena.org/page/FuncIntrface_NG911.

[29] NEXt Generation Emergency Services|NEXES Project|H2020|CORDIS|European Commission [Internet]. https://cordis.europa.eu/project/id/653337.

[30] EENA EENA NG112 Project [Internet]. https://eena.org/eena-ng112-project.

[31] Liberal F., Fajardo J.O., Lumbreras C., Kampichler W. (2017). European NG112 Crossroads: Toward a new emergency communications framework. IEEE Commun. Mag..

[32] Šterk M., Praprotnik M. (2017). Improving emergency response logistics through advanced GIS. Open Geospat. Data Softw. Stand..

[33] Osebor I., Misra S., Omoregbe N., Adewumi A., Fernandez-Sanz L. (2017). Experimental simulation-based performance evaluation of an SMS-based emergency geolocation notification system. J. Healthc. Eng..

[34] EMYNOS nExt generation eMergencY commuNicatiOnS H2020 project [Internet]. https://www.emynos.eu.

[35] Rescuetrack Advances Mobile Location [Internet]. http://www.rescuetrack.de/de-de/products/aml/.

[36] EENA Advanced Mobile Location [Internet]. https://eena.org/our-work/eena-special-focus/advanced-mobile-location/.

[37] Chikani V., Blust R., Vossbrink A., Wightman P., Bissell S., Graw J., Martinez R., Fisher B. (2020). Improving the continuum of care by bridging the gap between prehospital and hospital discharge data through stepwise deterministic linkage. Prehospital Emerg. Care..

[38] Hagger-Johnson G., Harron K., Goldstein H., Aldridge R., Gilbert R. (2017). Probabilistic linkage to enhance deterministic algorithms and reduce data linkage errors in hospital administrative data. J. Innov. Health Inform..

[39] Oostema J.A., Nickles A., Reeves M.J. (2020). A comparison of probabilistic and deterministic match strategies for linking prehospital and in-hospital stroke registry data. J. Stroke Cerebrovasc. Dis..

[40] Hughes-Gooding T., Dickson J.M., O’Keeffe C., Mason S.M. (2020). A data linkage study of suspected seizures in the urgent and emergency care system in the UK. Emerg. Med. J..

[41] Govindarajan P., Cook L., Ghilarducci D., Johnston C. (2012). Probabilistic matching of computerized emergency medical services records and emergency department and patient discharge data: A novel approach to evaluation of prehospital stroke care. Acad. Emerg. Med..

[42] Deserno T.M., Jakob R. Accident emergency informatics: Terminologies and standards are needed for digital health in the early rescue chain. Proceedings of the 2020 IEEE 14th International Conference on Application of Information and Communication Technologies (AICT).

[43] Cui J., Shao L., Zhong H., Xu Y., Liu L. (2018). Data aggregation with end-to-end confidentiality and integrity for large-scale wireless sensor networks. Peer-To-Peer Netw. Appl..

[44] SOG-IS Crypto Working Group Cryptographic Mechanisms [Internet]. https://www.sogis.eu/documents/cc/crypto/obsolete/SOGIS-Agreed-Cryptographic-Mechanisms-1.1.pdf.

[45] Informationstechnik Bundesamt für Sicherheit in der.BSI TR-03116-1 [Internet]. https://www.bsi.bund.de/SharedDocs/Downloads/DE/BSI/Publikationen/TechnischeRichtlinien/TR03116/BSI-TR-03116.pdf?__blob=publicationFile&v=3.

[46] Haghi M., Danyali S., Ayasseh S., Wang J., Aazami R., Deserno T.M. (2021). Wearable devices in health monitoring from the environmental towards multiple domains: A survey. Sensors.

[47] Langley A., Hamburg M., Turner S. (2016). Elliptic curves for security. RFC.

[48] Tan K.K., Goh H.L. Session Initiation Protocol. Proceedings of the IEEE International Conference on Industrial Technology.

[49] Li N. Research on Diffie-Hellman key exchange protocol. Proceedings of the ICCET 2010—2nd International Conference on Computer Engineering and Technology.

[50] Niasar M.B., El Khatib R., Azarderakhsh R., Mozaffari-Kermani M. Fast, small, and area-time efficient architectures for key-exchange on Curve25519. Proceedings of the 27th Symposium on Computer Arithmetic.

[51] Bisheh-Niasar M., Azarderakhsh R., Mozaffari-Kermani M. (2021). Cryptographic accelerators for digital signature based on Ed25519. IEEE Trans. Very Large Scale Integr. Syst..

[52] EENA Project NG112 and the New Emergency Services Networks Landscape Challenges and Opportunities [Internet]. https://eena.org/document/ng112-and-the-new-emergency-services-networks-landscape/.

[53] Barakat R., Deserno T.M., Deserno T.M., Chen P.-H. (2020). Automatic alerting of accidents and emergencies: The international standard accident number and vital sign data embedded in future PACS. Medical Imaging 2020: Imaging Informatics for Healthcare, Research, and Applications.

